# Coping Capacity and Episodic Memory in Older Adults With SMCs: The Mediating Role of Healthy Lifestyle

**DOI:** 10.1093/geroni/igaa057.891

**Published:** 2020-12-16

**Authors:** Feilong Wang, Shijie Li, Kaifa Wang, Yanni Yang

**Affiliations:** 1 Third Military Medical University, Chongqing, China; 2 Southwest University, Chongqing, China

## Abstract

Older adults with subjective memory complaints (SMCs) are at increased risk for episodic memory decline. Episodic memory decline is an important predictor of objective memory impairment (one of the earliest symptoms of Alzheimer’s disease) and an often-suggested criterion of successful memory aging. Therefore, it is important to explore the determinant factors that influence episodic memory in older adults with SMCs. Roy adaptation model and preliminary evidence suggest that older adults with SMCs undergo a coping and adaptation process, a process influenced by many health-related risks and protective factors. This study aimed to explore the relationship between coping capacity and episodic memory, and the mediating role of healthy lifestyle between coping capacity and episodic memory in a sample of 309 community-dwelling older adults with SMCs. Results from the structural equation modeling showed that coping capacity directly affects episodic memory (r=0.629, p＜0.001), and there is a partial mediating effect (60.5%) of healthy lifestyle among this sample of older adults with SMCs. This study demonstrates that coping capacity and adaptation positively correlate with episodic memory in older adults with SMCs, and that these correlations are mediated by healthy lifestyle. The results suggest that older adults with poor coping capacity should be assessed and monitored regularly, and clear lifestyle-related interventions initiated by healthcare providers that promote healthy lifestyles may effectively improve coping capacity and episodic memory in this population group. Note: First author: Feilong Wang, Co-first author: Shijie li, Corresponding author: Yanni Yang

